# Open-label extension of a randomized trial investigating safety and efficacy of rhPTH(1–84) in hypoparathyroidism

**DOI:** 10.1093/jbmrpl/ziad010

**Published:** 2024-01-05

**Authors:** Aliya A Khan, Lisa G Abbott, Intekhab Ahmed, Olulade Ayodele, Claudia Gagnon, Richard D Finkelman, Emese Mezosi, Lars Rejnmark, Istvan Takacs, Shaoming Yin, Steven W Ing

**Affiliations:** Divisions of Endocrinology and Metabolism and Geriatric Medicine, McMaster University, Hamilton, Ontario L8S 4L8, Canada; Northern Nevada Endocrinology, Reno, NV 89511, United States; University of Nevada, Reno, NV 89557, United States; Department of Endocrinology and Metabolism, Thomas Jefferson University Hospital, Philadelphia, PA 19107, United States; Takeda Development Center Americas Inc., Lexington, MA, 02421, United States; Department of Medicine, CHU de Québec-Université Laval Research Centre, Quebec G1V 4G2, Canada; Department of Medicine, Université Laval, Quebec G1V 0A6, Canada; Takeda Pharmaceuticals USA, Inc., Lexington, MA 02421, United States; Department of Internal Medicine, University of Pécs, 7624 Pécs, Hungary; Department of Clinical Medicine – Department of Endocrinology and Internal Medicine, Aarhus University, 8200, Aarhus, Denmark; Department of Internal Medicine and Oncology, Semmelweis University, 1083 Budapest, Hungary; Takeda Development Center Americas Inc., Lexington, MA, 02421, United States; Division of Endocrinology, Diabetes, and Metabolism, Ohio State University, Columbus, OH 43210, United States

**Keywords:** parathyroid-related disorders, biochemical markers of bone turnover, hormone replacement/receptor modulators, clinical trials, other (diseases and disorder of/related to bone)

## Abstract

Hypoparathyroidism (HypoPT) is a rare disease, often inadequately controlled by conventional treatment. PARALLAX was a mandatory post-marketing trial assessing pharmacokinetics and pharmacodynamics of different dosing regimens of recombinant human parathyroid hormone 1–84 (rhPTH[1–84]) for treating HypoPT. The present study (NCT03364738) was a phase 4, 1-yr open-label extension of PARALLAX. Patients received only 2 doses of rhPTH(1–84) in PARALLAX and were considered treatment-naive at the start of the current study. rhPTH(1–84) was initiated at 50 μg once daily, with doses adjusted based on albumin-corrected serum calcium levels. Albumin-corrected serum calcium (primary outcome measure), health-related quality of life (HRQoL), adverse events, and healthcare resource utilization (HCRU) were assessed. The mean age of the 22 patients included was 50.0 yr; 81.8% were women, and 90.9% were White. By the end of treatment (EOT), 95.5% of patients had albumin-corrected serum calcium values in the protocol-defined range of 1.88 mmol/L to the upper limit of normal. Serum phosphorus was within the healthy range, and albumin-corrected serum calcium-phosphorus product was below the upper healthy limit throughout, while mean 24-h urine calcium excretion decreased from baseline to EOT. Mean supplemental doses of calcium and active vitamin D were reduced from baseline to EOT (2402–855 mg/d and 0.8–0.2 μg/d, respectively). Mean serum bone turnover markers, bone-specific alkaline phosphatase, osteocalcin, procollagen type I N-terminal propeptide, and type I collagen C-telopeptide increased 2–5 fold from baseline to EOT. The HCRU, disease-related symptoms and impact on HRQoL improved numerically between baseline and EOT. Nine patients (40.9%) experienced treatment-related adverse events; no deaths were reported. Treatment with rhPTH(1–84) once daily for 1 yr improved HRQoL, maintained eucalcemia in 95% of patients, normalized serum phosphorus, and decreased urine calcium excretion. The effects observed on urine calcium and the safety profile are consistent with previous findings.

**Clinical trial identifier:**

NCT03364738.

## Introduction

Hypoparathyroidism (HypoPT) is a rare condition characterized by insufficient levels of parathyroid hormone (PTH), resulting in hypocalcemia, hyperphosphatemia, and hypercalciuria.^1^^,^^2^ The PTH plays a critical role in the regulation of serum calcium and phosphorus.^3^ As such, imbalances in calcium and phosphorus homeostasis caused by HypoPT may result in multisystem dysfunction including: neurologic and neuromuscular symptoms such as muscle cramps, paresthesia, and seizures^4^; renal complications with hypercalciuria, nephrocalcinosis, nephrolithiasis, and renal impairment^4^; respiratory complications such as bronchospasm and laryngospasm^4^; and cardiac complications such as bradyarrhythmias and congestive heart failure.^5^

Conventional treatment with oral calcium and active vitamin D often fails to adequately control HypoPT and does not normalize quality of life (QoL).^6^ In addition, there are concerns surrounding the increased risk of ectopic calcification and long-term complications.^7^^,^^8^ Patients with chronic HypoPT remain at a higher risk of hypercalciuria and renal complications, cardiovascular conditions, infections, and immune system impairment than the general population.^9-16^

The 24-wk pivotal Phase 3 REPLACE study (NCT00732615) found that once daily (QD) recombinant human parathyroid hormone 1–84 (rhPTH[1–84]) at 50, 75, or 100 μg was well tolerated and efficacious in treating adults with chronic HypoPT.^17^ Based on these data, rhPTH(1–84) was approved in 2015 as an adjunct to calcium and vitamin D to control hypocalcemia in patients with HypoPT in the USA.^18^ As a condition of the approval of rhPTH(1–84) for HypoPT, the US Food and Drug Administration (FDA) mandated the execution of a post-marketing trial to assess safety and pharmacokinetic and pharmacodynamic effects of alternative rhPTH(1–84) dosing regimens.^19^

To meet this requirement, the Phase 1 PARALLAX study (NCT02781844) assessed treatment with rhPTH(1–84) 25 μg twice a day (BID), 50 μg BID, or 100 μg QD, with and without supplemental calcium, on pharmacokinetic and pharmacodynamic outcomes over 24 h in adults with chronic HypoPT.^20^^,^^21^ PARALLAX revealed no differences thought to be clinically meaningful in pharmacokinetic or pharmacodynamic parameters between BID and QD rhPTH(1–84) dosing.^21^ Because PARALLAX excluded administration of PTH, fragments or analogs within 3 months^20^ and rhPTH(1–84) was only given for 2 single days on study, patients who completed PARALLAX were considered to be treatment-naive.

The present study was a phase 4, 1-yr open-label extension of PARALLAX that aimed to evaluate the safety and efficacy of 52 wk of rhPTH(1–84) QD in patients who had previously been exposed to rhPTH(1–84) in PARALLAX.

## Materials and methods

### Study design and participants

This was a 52-wk open-label study (ClinicalTrials.gov: NCT03364738) conducted at 10 sites in Canada, Denmark, Hungary, and the USA between September 26, 2018, and April 14, 2020. The study comprised a screening period, a 52-wk treatment period, and a 30-d safety follow-up with end of study contact (Supplementary Figure S1). For patients who terminated the study early, the end of treatment (EOT) measurements are presented at wk 52/EOT.

The study enrolled only adult patients with chronic HypoPT who had previously completed the PARALLAX study; therefore, sample size and power considerations were not applicable to the present study.

In brief, inclusion criteria were: age 18–85 yr, completion of PARALLAX, and the ability to provide consent to the study including a 30-d follow-up period. Exclusion criteria included: use of any other investigational study drug in the 3 months before the screening visit; presence of a significant disorder making the patient unsuitable for the study; treatment with PTH, PTH analog, or PTH fragment in the 30 d before the screening visit, and increased baseline risk for osteosarcoma. Full inclusion and exclusion criteria are provided in Supplementary Table S1.

### Treatment

All patients received rhPTH(1–84); treatment was initiated in the form of a 50 μg QD subcutaneous (SC) injection in the thigh performed either by the patient or a designee. If albumin-corrected serum calcium was >2.25 mmol/L (> 9.0 mg/dL), a starting dose of rhPTH(1–84) 25 μg was considered. The dose of rhPTH(1–84) could be increased in increments of 25 μg up to a maximum of 100 μg QD, no more frequently than every 2–4 wk, with the goal of achieving or maintaining albumin-corrected serum calcium levels in the range of 2.0–2.25 mmol/L (8.0–9.0 mg/dL). If needed, the rhPTH(1–84) dose could also be adjusted down at any time to avoid hypercalcemia or to mitigate any safety concerns. The protocol indicated that once a patient had achieved a stable albumin-corrected serum calcium concentration (2.0–2.25 mmol/L [8.0–9.0 mg/dL]) and had minimized supplemental doses of calcium and active vitamin D, they continued to receive that dose of rhPTH(1–84).

### Endpoints

The primary efficacy endpoint was the proportion of patients who achieved albumin-corrected serum calcium values in the range of 1.875 mmol/L (7.5 mg/dL) to the protocol-defined upper limit of normal (2.62 mmol/L [10.5 mg/dL]) at wk 24 and 52. In patients with HypoPT, a target range below the normal reference range is recommended, which could help to decrease the risk of hypercalcemia and hypercalciuria^22^^,^^23^; 1.9 mmol/L (7.5 mg/dL) is often considered a threshold for acute symptoms^4^^,^^24^ and thus was chosen as the lower end of the target range in this study.

Secondary endpoints were the changes from baseline to wk 4, 8, 16, 24, 32, 40, and 52 (EOT) in albumin-corrected serum calcium concentration, serum phosphorus concentration, albumin-corrected serum calcium-phosphorus product, and changes from baseline to wk 16, 32, and 52 (EOT) in 24-h urine calcium excretion, as well as percentage changes from baseline in prescribed supplemental oral calcium dose, prescribed supplemental active vitamin D dose, and serum bone turnover markers.

Ranges/upper limits for different parameters according to the literature are as follows: serum phosphorus level, 0.8–1.5 mmol/L (2.5–4.6 mg/dL); serum calcium-phosphorus product, below 4.4 mmol^2^/L^2^ (<55.0 mg^2^/dL^2^)^23^; urinary calcium excretion upper limit for men, 7.5 mmol/24 h (300 mg/24 h) and for women, 6.25 mmol/24 h (250 mg/ 24 h)^23^^,^^25^; bone-specific alkaline phosphatase (BAP), 44.0 U/L for men, 29.0 U/L for premenopausal women, and 75.7 U/L for postmenopausal women^26^; osteocalcin healthy reference range, 3–14 μg/L^27^; procollagen type I N-terminal propeptide (PINP) healthy range for men between 25 and 70 yr, 15–80 μg/L, and for women between 50 and 69 yr, 15–75 μg/L^28^; type I collagen C-telopeptide (CTX) healthy ranges for men between 40 and 60 yr, 130–600 ng/L and for women aged 50 or above, 100–700 ng/L.^25^^,^^28^

Exploratory endpoints included changes from baseline to wk 24 and 52 in HypoPT-related symptoms, health-related quality of life (HRQoL), and measures of healthcare resource utilization (HCRU) including frequency of encounters (outpatient visits, laboratory tests, and non-protocol scheduled procedures; emergency department visits; hospitalizations), length of stay, and reasons for the encounters. Safety analyses were performed to evaluate adverse events (AEs), laboratory data, and other clinical measures.

### Assessments

Serum calcium, albumin, phosphorus, magnesium, and 25-hydroxyvitamin D were measured at each study visit; 1,25-dihydroxyvitamin D was measured at screening, baseline, wk 24, and EOT. Twenty-four-hour urine collections were performed at baseline, wk 16, wk 32, and EOT.

Serum bone turnover markers including BAP, PINP, CTX, and total osteocalcin were measured at baseline, wk 8, wk 24, and EOT.

Patient-reported outcome assessments included the Hypoparathyroidism Symptom Diary (HypoPT-SD) questionnaire (7-d recall version), completed by each patient at all study visits from baseline to EOT; the EQ-5D-5L VAS questionnaire, completed by each patient at baseline, wk 24, and EOT; and the Patient Global Impression of Severity (PGI-S), completed by each patient at all visits from baseline to EOT.

The HypoPT-SD is a 13-item questionnaire assessing HypoPT-related symptoms and impacts on life. The symptom subscale consists of items 1–7; item 8 assesses anxiety and item 9 assesses sadness or depression, and they are scored as individual items; the impact subscale consists of items 10–13. Symptom-focused items (items 1–9) are scored on a 5-point scale and impact-focused items (items 10–13) on a 3-point scale, with higher scores indicating worse symptoms and impacts associated with or due to HypoPT. There is no global score for all 13 items.^29^^,^^30^ The 7-d recall version of the HypoPT-SD was used in this study.

The EQ-5D-5L is a generic, multi-attribute, health-related HRQoL assessment composed of a descriptive system and a visual analog scale (VAS). The EQ-5D-5L descriptive system comprises 5 dimensions: mobility, self-care, usual activities, pain/discomfort, and anxiety/depression. Each dimension has 5 levels of severity: (1) no problems, (2) slight problems, (3) moderate problems, (4) severe problems, and (5) unable to do/extreme problems. The EQ-5D-5L VAS records the patient’s self-rated health state on a 100-point vertical VAS (0 = worst imaginable health state, 100 = best imaginable health state).^31^

The PGI-S is used to rate severity of different clinical conditions.^32^ In the present study, patients were asked to rate the severity of their current HypoPT-related symptoms on a 5-point scale comprising “No symptoms”, “Mild”, “Moderate”, “Severe”, and “Very severe”.

HCRU questionnaires were completed at every study visit from baseline to EOT.

Safety assessments included treatment-emergent AEs (TEAEs), clinical laboratory tests, vital signs, electrocardiograms, and anti-PTH antibodies. The AEs were coded using the Medical Dictionary for Regulatory Activities.

### Statistical analyses

Descriptive statistics were used to summarize baseline characteristics as well as continuous and categorical variables: continuous variables were summarized by number of patients, mean, SD, median, minimum, and maximum; categorical and count variables were summarized by number of patients and percentage. For specified variables, 95% CIs were calculated.

Responses to the HCRU questionnaire were summarized descriptively over time, by visit. For patients who were in hospital overnight, the total duration of hospitalization (d) was calculated as the sum of all hospitalization durations at each scheduled visit. The average duration of hospitalizations (d) was calculated as the mean duration of hospitalization per patient. The total and average durations of hospitalizations were considered to be 0 for patients who were not hospitalized overnight at the time of the corresponding visit.

Descriptive analyses were performed using SAS software version 9.3 or newer (SAS Institute Inc., Cary, NC, USA). *Post hoc* linear mixed effects models were performed using biochemical, bone turnover and PRO data with “time” as the independent variable (fixed effects), “outcome measure” as the dependent variable (eg QoL), and “patient” as the random effect. The default compound symmetry covariance structure was used.

### Ethical considerations

This study was conducted in accordance with International Council for Harmonization of Technical Requirements for Pharmaceuticals for Human Use of Good Clinical Practice, the principles of the Declaration of Helsinki, and other applicable local ethical and legal requirements.

## Results

### Patient characteristics

**Figure 1:**
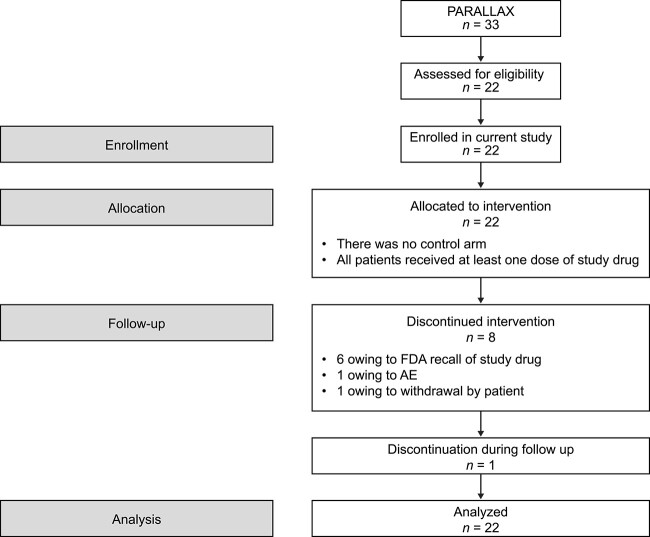
Patient flow in the study. All patients received rhPTH(1–84) and no randomization occurred. AE = adverse event; FDA = US Food and Drug Administration; rhPTH(1–84) = recombinant human parathyroid hormone (1–84).

All patients who completed the PARALLAX trial (*n* = 33) were offered an opportunity to enroll in the present follow-up, open-label extension trial. All 22 patients who consented and were screened were enrolled. Eight patients (36.4%) did not complete the study: 6 patients (27.3%) discontinued owing to the FDA-mandated recall of rhPTH(1–84),^33^ and one each (4.5%) discontinued because of AEs and personal choice (Figure 1). No patient was lost to follow-up or withdrew owing to lack of efficacy, and there were no protocol deviations that led to withdrawal.

Patients were predominately women (81.8%) and most patients were White (90.9%). One patient (4.5%) was Black or African American and one patient (4.5%) was Native American and Caucasian. The mean (SD) age of enrolled patients was 50.0 (11.4) yr and the mean (SD) body mass index was 31.5 (8.7) kg/m^2^. Patients had HypoPT for a mean (SD) of 10.3 (10.2) yr at study entry and the mean (SD) length of time between PARALLAX and the present study was 0.5 (0.5) yr (Table 1). Of the 22 patients who were enrolled, 19 (86.4%) were treated with calcitriol and 2 (9.1%) were treated with alfacalcidol, at mean (SD) doses of 1.0 (0.4) μg and 2.3 (1.1) μg, respectively. Data could not be calculated for one patient because of missing dose frequency information.

**Table 1:** Patient characteristics at baseline.

**Characteristic**	**Patients, *N* = 22**
Age, yr	
Mean (SD)	50.0 (11.4)
Median (range)	47.5 (28–71)
Sex, *n* (%)	
Female	18 (81.8)
Ethnicity, *n* (%)	
Hispanic or Latino	0
Not Hispanic or Latino	21 (95.5)
Not reported/unknown	1 (4.5)
Race, *n* (%)	
Black or African American	1 (4.5)
White	20 (90.9)
Other (Native American and Caucasian)	1 (4.5)
BMI, kg/m^2^	
Mean (SD)	31.5 (8.65)
Median (range)	30.4 (20.8–58.6)
Duration of HypoPT at study baseline (*n* = 21)^a^	
Yr, mean (SD)	10.3 (10.2)
Yr, median (range)	7.5 (1.7–39.0)

a For one patient no start date for HypoPT is available.

Abbreviation: HypoPT = hypoparathyroidism.

Of the 22 enrolled patients, 4 (18.2%) started rhPTH(1–84) at 25 μg and 18 (81.8%) started at 50 μg daily. At EOT, 5 patients (22.7%) took 25 μg, 8 patients (36.4%) 50 μg, 5 patients (22.7%) 75 μg, and 4 patients (18.2%) 100 μg daily.

### Efficacy evaluations

**Figure 2:**
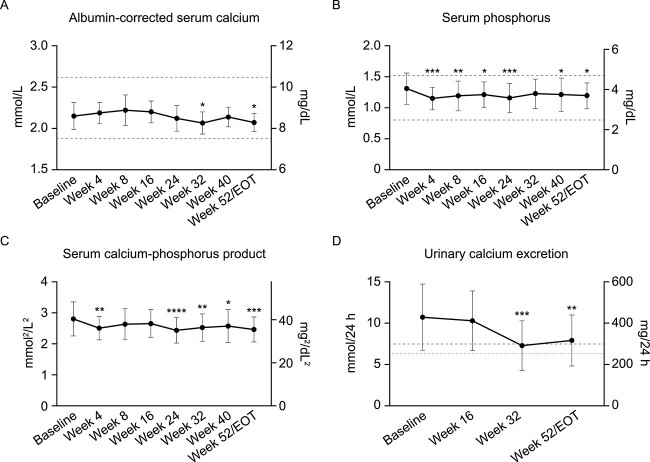
Mean (A) albumin-corrected serum calcium, (B) serum phosphorus, (C) albumin-corrected serum calcium-phosphorus product, and (D) 24-h urinary calcium excretion from baseline to wk 52. Data are means and SD. (A–C) Dashed lines indicate the protocol-defined primary endpoint range of (A) albumin-corrected serum calcium concentration: 1.875–2.62 mmol/L (7.5–10.5 mg/dL) and approximate normal ranges for (B) serum phosphorus level: 0.8–1.5 mmol/L (2.5–4.6 mg/dL),^34^ (C) serum calcium-phosphorus product: Below 4.4 mmol^2^/L^2^ (55 mg^2^/dL^2^).^23^ (D) Reference lines indicate upper limit for urinary calcium excretion level in men: 7.5 mmol/24 h (300 mg/24 h, dashed line) and women 6.25 mmol/24 h (250 mg/24 h, dotted line).^23^^,^^25^ Baseline is defined as the last available predose value. For patients who terminated the study early, the EOT measurements are presented at wk 52/EOT. *N* = 22 at baseline, *n* = 21 at wk 4 to wk 24, *n* = 20 at wk 32, *n* = 17 at wk 40, and *n* = 22 at wk 52/EOT (except urinary calcium: *N* = 20 at wk 52/EOT). *P-*values from *post hoc* analysis of change from baseline to each timepoint: ^*^P < .05, ^*^^*^P < .01, ^*^^*^^*^P < .001, ^*^^*^^*^^*^P < .0001. EOT = end of treatment.

At wk 24 and 52, the proportions of patients with albumin-corrected serum calcium values in the protocol-defined target range of 1.875–2.62 mmol/L (7.5–10.5 mg/dL) were 100.0% (21/21; 95% CI 84–100) and 95.5% (21/22, 95% CI 77–100), respectively. Mean (SD) albumin-corrected serum calcium concentrations were 2.1 (0.2) mmol/L (8.6 [0.7] mg/dL) at baseline and 2.1 (0.1) mmol/L (8.3 [0.4] mg/dL) at EOT (*P* = .0395, Figure 2A). Serum phosphorus was maintained within the reference range (0.8–1.5 mmol/L [2.5–4.7 mg/dL]),^34^ with a mean (SD) of 1.3 (0.3) mmol/L (4.0 [0.8] mg/dL) at baseline and of 1.2 (0.2) mmol/L (3.7 [0.6] mg/dL) at EOT (*P* = .0106, Figure 2B). Mean (SD) albumin-corrected serum calcium-phosphorus product was 2.8 (0.6) mmol^2^/L^2^ (34.7 [6.8] mg^2^/dL^2^) at baseline and 2.5 (0.4) mmol^2^/L^2^ (30.5 [5.0] mg^2^/dL^2^) at EOT (*P* = .0003, Figure 2C).

Mean (SD) 24-h urine calcium excretion was 10.7 (4.0) mmol/24 h (427.4 [160.0] mg/24 h) at baseline and 7.9 (3.1) mmol/24 h (315.8 [123.2] mg/24 h) at EOT (*P* = .0018, Figure 2D). The proportions of patients with 24-h urine calcium excretion within the normal range at baseline and EOT were 13.6% (3/22) and 35.0% (7/20), respectively. Only one patient’s 24-h urine calcium excretion was within the normal range at both baseline and EOT.

Mean (SD) prescribed daily oral supplemental calcium dose decreased by 64% from a baseline of 2402 (2350) mg/d to 855 (759) mg/d at EOT. Mean (SD) daily supplemental active vitamin D dose decreased by 75%, from 0.8 (0.4) μg/d at baseline to 0.2 (0.3) μg/d at EOT. The proportions of patients still being prescribed calcium and active vitamin D supplements at EOT were 72.7% and 31.8%, respectively, with 31.8% of patients still being prescribed both supplements. At EOT, one patient (4.6%) was prescribed active vitamin D at a dose of ˂0.25 μg/d, and one patient was prescribed calcium at ˂500 mg/d.

**Figure 3:**
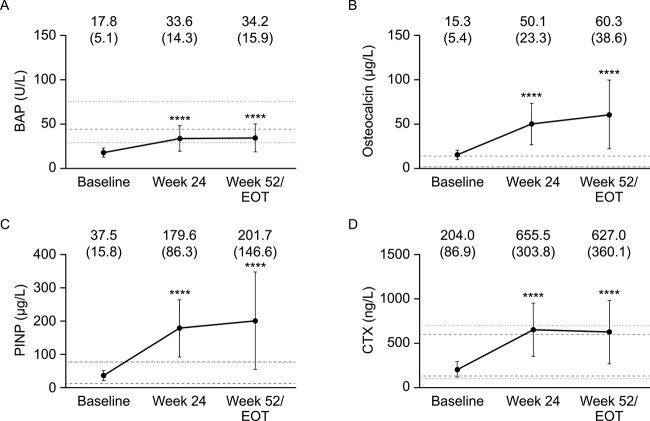
Mean (A) bone-specific alkaline phosphatase, (B) osteocalcin, (C) procollagen type I N-terminal propeptide, and (D) type I collagen C-telopeptide at baseline, wk 24, and wk 52/EOT. Data are mean (SD). Lines indicate reference ranges or values for healthy people. (A) <44.0 U/L for men (dashed line), <29.0 U/L for premenopausal women, and < 75.7 U/L for postmenopausal women (dotted lines)^26^; (B) healthy reference range (dashed lines): 3–14 μg/L^27^; (C) healthy range for men between 25 and 70 yr is 15–80 μg/L (dashed lines), and for women between 50 and 69 yr is 15–75 μg/L (dotted lines)^28^; (D) healthy range for men between 40 and 60 yr is 130–600 ng/L (dashed lines) and for women aged 50 or above is 100–700 ng/L (dotted lines).^28^*N* = 22 at baseline, *n* = 21 at wk 24, and *n* = 22 at wk 52/EOT. *P-*values from *post hoc* analysis of change from baseline to each timepoint: ^*^^*^^*^^*^P < .0001. BAP = bone-specific alkaline phosphatase; CTX = type I collagen C-telopeptide; EOT = end of treatment; PINP = procollagen type I N-terminal propeptide.

Overall, serum bone turnover marker concentrations were significantly higher at wk 24 than at baseline (Figure 3). An increase was observed between wk 24 and 52 for all markers except CTX which, although remaining higher than baseline and similar to healthy reference ranges, decreased slightly between wk 24 and 52 (Figure 3D). Mean BAP levels were lower than the normal range at baseline but within the reference range at wk 24 and EOT. Mean osteocalcin levels were similar to healthy median reference levels at baseline, but higher than the reference values at wk 24 and EOT. The same was observed for mean PINP.

### Healthcare resource utilization

The mean number of doctor or healthcare professional visits for any health reason during the past 12 months, and those due to hypocalcemia or other causes, decreased from baseline to wk 24 and 52. During the 12 months before baseline, one patient (4.5%) was hospitalized, and 4 patients (18.2%) had required an emergency department visit. The mean (SD) length of hospitalization was 1.0 (4.69) d at baseline. At wk 24, out of 21 patients with available data, no patient required hospitalization and one patient (4.8%) required an emergency department visit; at wk 52 no patient required hospitalization and 3 patients (13.6%) required an emergency department visit (Table 2). Two of the 3 patients who required an emergency department visit at EOT had also required an emergency department visit at baseline.

**Table 2:** Healthcare resource utilization at different time points.

**Time point**	**Number (%) of patients with any hospitalization**	**Number (%) of patients with any ED visit**	**Total duration of hospitalization,** d	
			**Mean (SD)**	**Range**
Baseline (*n* = 22)	1 (4.5)	4 (18.2)	1.0 (4.69)	0–22
Wk 4 (*n* = 21)	0	0	0	0
Wk 8 (*n* = 21)	0	1 (4.8)	0	0
Wk 16 (*n* = 21)	1 (4.8)	1 (4.8)	0.1 (0.65)	0–3
Wk 24 (*n* = 21)	0	1 (4.8)	0	0
Wk 32 (*n* = 20)	0	0	0	0
Wk 40 (*n* = 17)	1 (5.9)	0	0.2 (0.73)	0–3
Wk 52 (*n* = 22)	0	3 (13.6)	0	0

Abbreviations: ED = emergency department; *n* = number of patients with data available.

### Hypoparathyroidism symptoms and health-related quality of life

**Figure 4:**
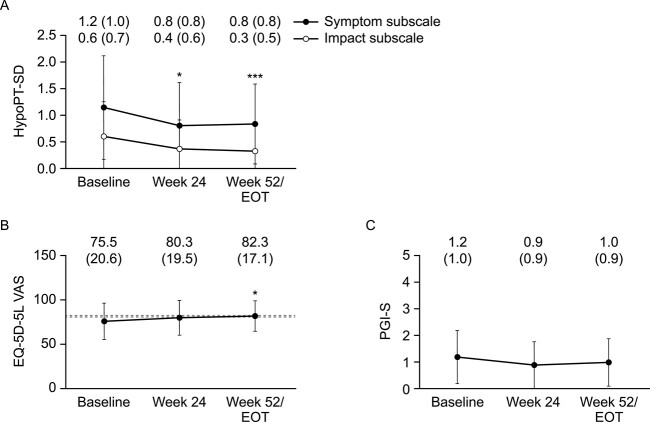
Mean (SD) (A) HypoPT-SD, (B) EQ-5D-5L-VAS, (C) PGI-S scores at baseline, wk 24, and wk 52/EOT. Data are mean (SD). Lines in (B) indicate the range of mean EQ-5D-5L VAS score for the general population in the USA (80.4),^47^ Canada (82.7),^48^ Hungary (81.6),^50^ and Denmark (82.4).^49^ HypoPT-SD: Higher symptom subscale scores indicate worse symptoms of HypoPT, and higher impact subscale scores indicate worse impacts from HypoPT. EQ-5D-5L VAS: Higher scores indicate better health-related quality of life. PGI-S severity was rated on a 5-point scale comprising “no symptoms”, “Mild”, “Moderate”, “Severe”, and “Very severe”. *N* = 22 at baseline, *n* = 21 at wk 24, and *n* = 22 at wk 52/EOT, except for HypoPT-SD impact subscale: EOT *n* = 21. *P-*values from *post hoc* analysis of change from baseline to each timepoint: ^*^P < .05, ^*^^*^^*^P < .001. EOT = end of treatment; HypoPT-SD = hypoparathyroidism symptom diary; PGI-S = patient global impression of severity; VAS = visual analog scale.

All measures of disease burden improved numerically between baseline and wk 52 (Figure 4). The mean (SD) HypoPT-SD symptom subscale score significantly decreased from 1.2 (1.0) at baseline to 0.8 (0.8), *P* = .0250, and 0.8 (0.8), *P* = .0169, at wk 24 and 52, respectively. The mean (SD) HypoPT-SD impact subscale score significantly decreased from 0.6 (0.7) at baseline to 0.4 (0.6), *P* = .0023, and 0.3 (0.5), *P* = .0005, at wk 24 and 52, respectively. The mean (SD) EQ-5D-5L VAS score increased from 75.5 (20.6) at baseline to 80.3 (19.5*), P* = .2937, and 82.3 (17.1), *P* = .0156, at wk 24 and 52, respectively. Severe mobility issues and problems in performing usual activities were experienced by 2 patients (9.1%) each at baseline and no patients by EOT; severe pain or discomfort was experienced by 3 patients (13.6%) at baseline and no patients by EOT; severe anxiety or depression was experienced by one patient (4.5%) at baseline and no patients by EOT. Mean (SD) PGI-S scores numerically decreased from 1.2 (1.0) at baseline to 0.9 (0.9) and 1.0 (0.9) at wk 24 and 52, respectively, although these decreases were not significant.

### Safety

Overall, 17 patients (77.3%) experienced a total of 163 TEAEs, of which 78 TEAEs experienced by 9 patients (40.9%) were considered related to rhPTH(1–84) (Table 3). The most common treatment-related AEs were nausea, fatigue, hypercalcemia, and headache. Four hypocalcemic and 5 hypercalcemic TEAEs were recorded: 2 and 3 of which, respectively, were considered treatment related. Three of the 4 hypocalcemic events occurred after wk 4 and before wk 16; the fourth event occurred around the wk 52 EOT visit.

**Table 3:** Treatment-emergent adverse events.

**Category**	**Patients, *N* = 22 (%)**	**Events**
Any TEAE	17 (77.3)	163
Hypocalcemic TEAE	2 (9.1)	4
Treatment-related TEAE	9 (40.9)	78
TEAE leading to withdrawal	1 (4.5)	2
TEAE by highest severity		
Mild	10 (45.5)	–
Moderate	4 (18.2)	–
Severe	3 (13.6)	–
Serious TEAE	4 (18.2)	4
Serious TEAE by highest severity		
Mild	1 (4.5)	–
Moderate	2 (9.1)	–
Severe	1 (4.5)	–
TEAE leading to death	0	–
System organ class^a^ Preferred term		
Infections and infestations	8 (36.4)	16
Nasopharyngitis	3 (13.6)	3
Gastrointestinal disorders	6 (27.3)	13
Nausea	6 (27.3)	7
Vomiting	2 (9.1)	4
Musculoskeletal and connective tissue disorders	6 (27.3)	19
Muscle spasms	3 (13.6)	6
Myalgia	2 (9.1)	2
Osteoarthritis	2 (9.1)	2
Tendonitis	2 (9.1)	2
Nervous system disorders	6 (27.3)	17
Headache	3 (13.6)	7
Migraine	2 (9.1)	3
Paresthesia	2 (9.1)	2
General disorders and administration site conditions	5 (22.7)	59
Fatigue	4 (18.2)	5
Metabolism and nutrition disorders	5 (22.7)	11
Hypercalcemia	4 (18.2)	5
Hypocalcemia	2 (9.1)	4
Psychiatric disorders	4 (18.2)	5
Anxiety	2 (9.1)	2

a Frequently occurring (in >5% of patients) TEAEs.

Abbreviation: TEAE = treatment-emergent adverse event.

One patient (4.5%) experienced 2 treatment-related AEs that resulted in study withdrawal (mild nausea and mild hypersensitivity, described as a generalized allergic reaction); both events resolved upon study drug discontinuation. No deaths occurred during the study.

Mean (SD) levels of 25-hydroxyvitamin D were 110.5 (28.56) nmol/L at baseline and 89.7 (21.16) and 88.5 (21.89) at wk 24 and 52, respectively.

## Discussion

This open-label extension study evaluated the safety and efficacy of 1 yr of rhPTH(1–84) treatment, administered once daily by SC injection to patients with HypoPT who had previously taken part in the PARALLAX study. Before participation in the present study, patients had received a maximum of 2 single doses of rhPTH(1–84) in PARALLAX, essentially rendering them naive to rhPTH(1–84). Furthermore, patients in PARALLAX were likely to have been naive to rhPTH(1–84) before enrollment, although, owing to the exclusion criteria, it is possible that patients may have received rhPTH(1–84) ˃3 months before the first dose within the study period. In consideration of the half-life of PTH, however,^35^ there would have been no residual effects of any exogenously administered PTH product at baseline.

The ability of rhPTH(1–84) to maintain not only albumin-corrected serum calcium, but also serum phosphorus, albumin-corrected serum calcium-phosphorus product, and 24-h urinary calcium excretion levels within normal ranges is important, particularly because conventional treatment for HypoPT often does not adequately achieve this, leaving patients at increased risk of renal complications.^9^ In a previous study, patients who received rhPTH(1–84) had a significantly lower risk of chronic kidney disease over a 5-yr period compared to a historical control cohort of patients with HypoPT who received conventional treatment.^36^

In the present study, the primary endpoint of albumin-corrected serum calcium values in the range of 1.875 mmol/L to the upper limit of normal was met by all patients at wk 24 and almost all patients (95.5%) at wk 52. Overall trends observed for biochemical parameters were consistent with those expected for efficacious treatment of HypoPT.^23^ Mean albumin-corrected serum calcium concentrations were maintained within the target range for patients with HypoPT and mean serum phosphorus concentrations decreased to within the healthy reference range. Mean 24-h urinary calcium excretion declined but was above the upper level of the reference interval at wk 52. These results are consistent with previous studies of long-term use of rhPTH(1–84).^37-39^

After rhPTH(1–84) initiation, we observed a numerical reduction in serum phosphorus similar to that observed within 4 wk of rhPTH(1–84) initiation in the previous open-label extension study, RACE (NCT01297309).^37^ This observed reduction may be in response to the increased phosphorus excretion that results from treatment with rhPTH(1–84).^40^

In the present study, the patients’ need for supplemental calcium and active vitamin D decreased numerically over time. Similar results were achieved in a previous trial, in which injections of rhPTH(1–84) at 100 μg every other day for 24 months significantly reduced requirement for supplementation.^41^

The PTH regulates bone remodeling^42^ and patients with HypoPT may have downregulated bone turnover,^43^ which is associated with increased bone mineral density,^44^ although it is unclear whether these changes affect risk of fracture.^45^ In the present study, serum bone turnover markers increased numerically from baseline to EOT, as was observed in a previous study of long-term treatment with rhPTH(1–84) in which markers reached a peak 1 yr after treatment initiation.^39^

Compared with healthy individuals, patients with HypoPT who receive conventional treatment with supplemental calcium or active vitamin D have been shown to report significantly higher levels of depression and anxiety.^46^ The descriptive statistics for the HypoPT-SD symptom subscale scores indicated that patients in the present study had mild to moderate symptom severity at baseline that decreased (improved) from baseline to EOT. However, meaningful within-patient change thresholds are yet to be determined.

According to published data, mean EQ-5D-5L VAS scores in the USA for “good health” are 75.8 and 77.2 for men and women, respectively, and 84.6 and 85.3 for “very good health” for men and women, respectively.^47^ The mean EQ-5D-5L VAS score for the general population has been reported as 82.7 in Canada,^48^ 82.4 in Denmark,^49^ 81.6 in Hungary,^50^ and 80.4 in the USA.^47^ In the current study, mean EQ-5D-5L VAS scores increased (improved) from a mean score of 75.5 at baseline, which was below that reported for the general population, to a mean value that was closer to reported values for the general population by EOT: 82.3. No notable changes from baseline were observed in the proportions of patients who reported moderate or more severe EQ-5D-5L responses at wk 24 and 52.

The mean PGI-S scores at wk 24, and at wk 52 were lower (improved) compared with baseline. Improvements in the HRQoL domains of lower bodily pain, general health, vitality, and mental health were also observed during 24 wk of rhPTH(1–84) treatment in the pivotal REPLACE study, as measured by the RAND 36-item Short Form Health Survey.^51^

The burden of HypoPT on healthcare systems is substantial. In England, HCRU was significantly higher among patients with chronic HypoPT than patients with hypothyroidism, and those who underwent thyroid surgery but did not have HypoPT.^52^ In the USA, annual medical costs for patients with HypoPT have been reported to be 3 times that of age- and sex-matched controls.^53^ In the present study, the mean number of doctor/healthcare professional visits decreased from baseline to wk 24 and 52.

The safety profile of rhPTH(1–84) was consistent with that reported in previous studies.^37^^,^^39^ No deaths occurred during the study and no serious TEAEs were considered treatment related; 3 hypercalcemic events were considered treatment related.

Although this study provides additional evidence on the efficacy and safety of rhPTH(1–84), as well as the impact on patient-reported outcomes, from a cohort of essentially treatment-naïve HypoPT patients in a setting representative of the real world, the study is subject to several limitations: the sample size was small, although this is reflective of the rarity of the disease^1^ and the fact that this was an extension study, and there was no control group with which to compare our results. Data on HypoPT etiology and the reasons for emergency visits were not available. Also, the period of observation was relatively short.

Owing to manufacturing challenges, production of rhPTH(1–84) will be discontinued by the end of 2024,^33^ nevertheless, the study adds to the existing evidence supporting the efficacy of rhPTH(1–84) and provides additional evidence on hormone replacement therapies in general for the treatment of HypoPT. Other PTH analogs with different pharmacokinetic or pharmacodynamic profiles are under development; at least one of these is currently under review by the FDA.

In conclusion, once-daily treatment with rhPTH(1–84) for 1 yr was efficacious in this population of patients with HypoPT; albumin-corrected serum calcium was maintained in the protocol-defined target range, and reductions were observed in oral calcium and active vitamin D requirements, serum phosphorus concentrations, and 24-h urine calcium excretion. The safety profile was consistent with previously reported findings.

## Supplementary Material

Study_404_supplemental_materials_revised_clean_ziad010

## Data Availability

The datasets, including the redacted study protocol, redacted statistical analysis plan, and individual participant’s data supporting the results reported in this article, will be made available within 3 months from initial request, to researchers who provide a methodologically sound proposal. The data will be provided after its de-identification, in compliance with applicable privacy laws, data protection and requirements for consent and anonymization.
