# Correction to: Open-label extension of a randomized trial investigating safety and efficacy of rhPTH(1–84) in hypoparathyroidism

**DOI:** 10.1093/jbmrpl/ziae068

**Published:** 2024-05-17

This is a correction to: Aliya A Khan, Lisa G Abbott, Intekhab Ahmed, Olulade Ayodele, Claudia Gagnon, Richard D Finkelman, Emese Mezosi, Lars Rejnmark, Istvan Takacs, Shaoming Yin, Steven W Ing, Open-label extension of a randomized trial investigating safety and efficacy of rhPTH(1–84) in hypoparathyroidism, *JBMR Plus*, Volume 8, Issue 3, March 2024, ziad010, https://doi.org/10.1093/jbmrpl/ziad010.

Affiliation information has been updated to reflect that Takeda Development Center Americas Inc. is located in Cambridge, MA, and that the tenth-listed author is affiliated with Takeda Pharmaceuticals USA, Inc. rather than Takeda Development Center Americas Inc.

