# Supplementary material for: Open-label extension of a randomized trial investigating safety and efficacy of rhPTH(1–84) in hypoparathyroidism
Source: JBMR Plus. 2024 Jan 5;8(3):ziad010. doi: 10.1093/jbmrpl/ziad010 (PMC11090130; doi:10.1093/jbmrpl/ziad010)
Supplement: Study_404_supplemental_materials_revised_clean_ziad010 [file study_404_supplemental_materials_revised_clean_ziad010.pdf]

Supplementary Table 1. Full Inclusion and Exclusion Criteria

Inclusion criteria
• 18–85 years of age<br>• Completion of PARALLAX, including follow-up<br>• Willingness and ability to comply with the study procedures and restrictions and provide informed consent for participation<br>• Men or nonpregnant, nonlactating women who comply with contraceptive requirements or are of nonchildbearing potential
**Exclusion criteria**
• Treatment with any investigational product, aside from that received during the study, within 3 months prior to the screening visit<br>• Treatment with PTH, PTH analog, or PTH fragment within the last 30 days from the screening visit<br>• Use of the following medications prior to administration of rhPTH(1–84) within: 30 days – loop diuretics, lithium, systemic corticosteroids (as judged by the investigator: primarily high doses of systemic corticosteroids were to be excluded; stable doses of hydrocortisone may have been acceptable); 3 months – cinacalcet hydrochloride; 6 months – fluoride tablets, oral bisphosphonates, methotrexate, growth hormone, digoxin; 12 months – intravenous bisphosphonates, drug or alcohol abuse<br>• Clinically relevant issues or risk factors contraindicating study participation[a]<br>• History of clinically significant illness during the 4 weeks prior to dosing, or clinically significant procedure within 8 weeks of first dose, as determined by investigator, or expected to undergo a major surgical procedure during the study

- Diseases affecting calcium metabolism of calcium-phosphorus homeostasis other than HypoPT
- History of PTH intolerance, allergic response to PTH, PTH analogs or PTH(1–34) analogs or other significant allergies contraindicating study participation

[a]Includes patients who were at increased baseline risk for osteosarcoma, such as patients with Paget's disease of bone or unexplained elevations of alkaline phosphatase, young adult patients with open epiphyses, patients with hereditary disorders predisposing to osteosarcoma or patients with a prior history of external beam or implant radiation therapy involving the skeleton.
HypoPT = hypoparathyroidism; PTH = parathyroid hormone; rhPTH(1–84) = recombinant human parathyroid hormone (1–84).

Supplementary Table 2. *Post hoc* analyses mixed effects models of the difference from baseline in biomarkers (Fig 2), bone turnover markers (Fig 3), and HRQoL parameters (Fig 4)

Figure	Parameter	Overall *P* (ProbF)	Timepoint	Estimated difference from baseline	*P*	Lower limit	Upper limit
Fig 2A	Albumin-corrected serum calcium	0.0001	Week 4	0.04080	0.2756	-0.03290	0.1145
			Week 8	0.07223	0.0547	-0.00147	0.1459
			Week 16	0.05270	0.1596	-0.02100	0.1264
			Week 24	-0.02492	0.5049	-0.09862	0.04879
			Week 32	-0.08182	0.0321	-0.1566	-0.00708
			Week 40	-0.00545	0.8909	-0.08389	0.07298
			Week 52/EOT	-0.07636	0.0395	-0.1490	-0.00371
Fig 2B	Serum phosphorus	0.0172	Week 4	-0.1629	0.0004	-0.2510	-0.07483
			Week 8	-0.1201	0.0079	-0.2081	-0.03198
			Week 16	-0.1029	0.0224	-0.1910	-0.01483
			Week 24	-0.1562	0.0006	-0.2443	-0.06817
			Week 32	-0.08875	0.0516	-0.1781	0.000609
			Week 40	-0.1059	0.0272	-0.1998	-0.01208
			Week 52/EOT	-0.1136	0.0106	-0.2003	-0.02695
Fig 2C	Albumin-corrected serum calcium-phosphorus product	0.0021	Week 4	-0.3017	0.0015	-0.4862	-0.1172
			Week 8	-0.1784	0.0580	-0.3630	0.006114
			Week 16	-0.1543	0.1006	-0.3388	0.03025
			Week 24	-0.3759	<.0001	-0.5604	-0.1913
			Week 32	-0.2939	0.0023	-0.4811	-0.1067
			Week 40	-0.2391	0.0175	-0.4357	-0.04245
			Week 52/EOT	-0.3372	0.0003	-0.5188	-0.1555
Fig 2D	Urinary calcium excretion	0.0001	Week 16	-0.3446	0.6731	-1.9711	1.2820
			Week 32	-3.3037	0.0002	-4.9553	-1.6521
			Week 52/EOT	-2.6960	0.0018	-4.3476	-1.0444

Figure	Parameter	Overall *P* (ProbF)	Timepoint	Estimated difference from baseline	*P*	Lower limit	Upper limit
Fig 3A	BAP	<.0001	Week 24	15.6520	<.0001	10.6391	20.6649
			Week 52/EOT	16.4636	<.0001	11.5305	21.3968
Fig 3B	Osteocalcin	<.0001	Week 24	33.9526	<.0001	21.6909	46.2144
			Week 52/EOT	44.9273	<.0001	32.8512	57.0034
Fig 3C	PINP	<.0001	Week 24	139.74	<.0001	91.2803	188.20
			Week 52/EOT	164.25	<.0001	116.51	211.99
Fig 3D	CTX	<.0001	Week 24	445.02	<.0001	305.32	584.71
			Week 52/EOT	423.00	<.0001	285.34	560.66
Fig 4A	Symptom subscale	0.0281	Week 24	-0.2928	0.0250	-0.5469	-0.03870
			Week 52/EOT	-0.3081	0.0169	-0.5580	-0.05821
Fig 4A	Impact subscale	0.0009	Week 24	-0.2186	0.0023	-0.3543	-0.08291
			Week 52/EOT	-0.2543	0.0005	-0.3901	-0.1186
Fig 4B	EQ-5D-5L VAS	0.0510	Week 24	2.8848	0.2937	-2.5925	8.3621
			Week 52/EOT	6.7273	0.0156	1.3418	12.1128
Fig 4C	PGI-S	0.2898	Week 24	-0.2418	0.1193	-0.5487	0.06513
			Week 52/EOT	-0.1364	0.3669	-0.4382	0.1655

BAP = bone-specific alkaline phosphatase; CTX = type I collagen C-telopeptide; EOT = end of treatment; HRQoL= health-related quality of life; PGI-S = Patient Global Impression of Severity; PINP = procollagen type I N-terminal propeptide; VAS = visual analogue scale.

Supplementary Fig. 1. Study design.

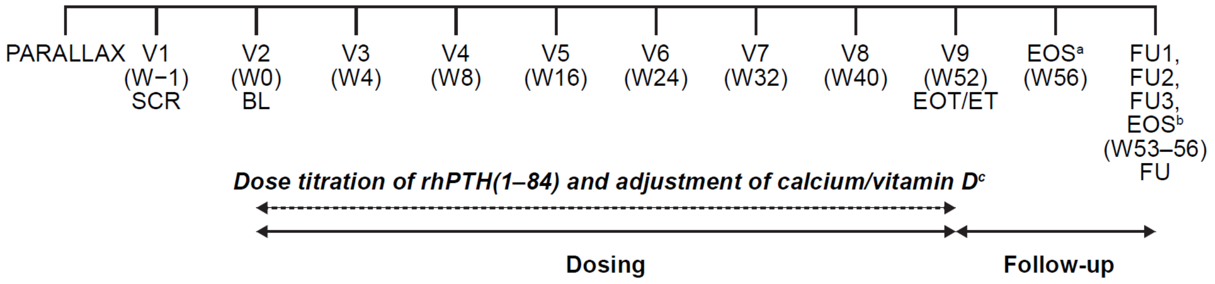

[a]Patients who transferred to commercial rhPTH(1–84) immediately after the EOT/ET visit (V9/W52) had an EOS (V10/W56) contact 30 days following the last dose of rhPTH(1–84). [b]Patients who did not continue on rhPTH(1–84) following the EOT/ET visit (V9) proceeded with weekly follow-up visits (FU1/W53, FU2/W54, and FU3/W55) with serum calcium measurements until a maximum of 30 days had elapsed. All patients had an EOS contact (V10/W56) 30 days after the final dose of study drug. Patients who transferred to commercial rhPTH(1–84) but experienced a treatment gap after the EOT/ET visit (V9/W52) proceeded with weekly follow-up visits (FU1/W53, FU2/W54, and FU3/W55) with serum calcium measurements until receiving commercial rhPTH(1–84) or until a maximum of 30 days had elapsed. All patients had an EOS contact (V10/W56) 30 days after the final dose of study drug. [c]Total serum calcium was measured 3–7 days after any adjustment of study drug and/or supplements. BL = baseline; EOT = end of treatment; EOS = end of study; ET = early termination; FU = follow-up; rhPTH(1–84) = recombinant human parathyroid hormone (1–84); SCR = screening; V = visit; W = week.
